# Effects of PM_2.5_ and gases exposure during prenatal and early-life on autism–like phenotypes in male rat offspring

**DOI:** 10.1186/s12989-020-0336-y

**Published:** 2020-01-29

**Authors:** Baharan Emam, Abbas Shahsavani, Fariba Khodagholi, Saeed Motesaddi Zarandi, Philip K. Hopke, Mostafa Hadei, Hamidreza Behbahani, Maryam Yarahmadi

**Affiliations:** 1 Department of Environmental Health Engineering, School of Public Health and Safety, Shahid Beheshti University of Medical Sciences, Tehran, Iran; 2 Environmental and Occupational Hazards Control Research Center, Shahid Beheshti University of Medical Sciences, Tehran, Iran; 3 Neuroscience Research Center, Shahid Beheshti University of Medical Sciences, Tehran, Iran; 4 Department of Public Health Sciences, University of Rochester School of Medicine and Dentistry, Rochester, NY 14642 USA; 5 Center for Air Resources Engineering and Science, Clarkson University, Potsdam, NY 13699 USA; 6 Department of Environmental Health Engineering, School of Public Health, Tehran University of Medical Sciences, Tehran, Iran; 7 Students’ Scientific Research Center (SSRC), Tehran University of Medical Sciences, Tehran, Iran; 8 Center of Environmental and Occupational health, Ministry of Health and Medical Education, Tehran, Iran

**Keywords:** Air pollution, Fine particulate matter, Behavioral assessment, OXTR protein

## Abstract

**Background:**

Epidemiological studies have reported associations between elevated air pollution and autism spectrum disorders (ASD). However, we hypothesized that exposure to air pollution that mimics real world scenarios, is a potential contributor to ASD. The exact etiology and molecular mechanisms underlying ASD are not well understood. Thus, we assessed whether changes in OXTR levels may be part of the mechanism linking PM_2.5_/gaseous pollutant exposure and ASD. The current in-vivo study investigated the effect of exposure to fine particulate matter (PM_2.5_) and gaseous pollutants on ASD using behavioral and molecular experiments. Four exposure groups of Wistar rats were included in this study: 1) particulate matter and gaseous pollutants exposed (PGE), 2) gaseous pollutants only exposed (GE), 3) autism-like model (ALM) with VPA induction, and 4) clean air exposed (CAE) as the control. Pregnant dams and male pups were exposed to air pollutants from embryonic day (E0) to postnatal day (PND21).

**Results:**

The average ± SD concentrations of air pollutants were: PM_2.5_: 43.8 ± 21.1 μg/m^3^, CO: 13.5 ± 2.5 ppm, NO_2_: 0.341 ± 0.100 ppm, SO_2_: 0.275 ± 0.07 ppm, and O_3_: 0.135 ± 0.01 ppm. The OXTR protein level, catalase activity (CAT), and GSH concentrations in the ALM, PGE, and GE rats were lower than those in control group (CAE). However, the decrements in the GE rats were smaller than other groups. Also in behavioral assessments, the ALM, PGE, and GE rats demonstrated a repetitive /restricted behavior and poor social interaction, but the GE rats had weaker responses compared to other groups of rats. The PGE and GE rats showed similar trends in these tests compared to the VPA rats.

**Conclusions:**

This study suggested that exposure to ambient air pollution contributed to ASD and that OXTR protein may serve as part of the mechanism linking them.

## Background

Autism spectrum disorder (ASD) is a pervasive neurodevelopmental disorder recognized by social communication deficits and restricted/repetitive patterns of behavior [1]. It is estimated that the global prevalence of ASD is 1 in 132 persons [2] and the prevalence rate is still increasing [3]. The prevalence of ASD is four to five times higher in males than females [4]. ASD has attracted public attention because of its high social costs and substantial impacts on society [5]. Although genetics likely plays an important role in ASD, environmental exposures to pollutants particularly during the early life periods could be another potential risk factor [6, 7]. Environmental factors such as exposure to air pollution may contribute to ASD etiology [8–10].

Previous studies point to a biological pathway linked to autism through a systemic inflammatory response that can affect the development of the central nervous system [9]. Developmental exposure to traffic-related air pollution (TRAP) has been associated with increased ASD risk [11]. Environment exposures during perinatal and postnatal periods may be crucial in ADS since brain development takes place in these periods, and exposure to environmental chemicals may cause neurodevelopmental disorders [12, 13].

Limited prior animal studies also suggested a connection between exposure to air pollution and ASD [14]. Most of these studies exposed rats or mice to high concentrations of air pollution. For instance, in a study was conducted by Li et al. (2018), rats were exposed to PM_2.5_ with doses of 2 or 20 mg/kg body weight per day [9], and reported that both groups of exposed rats showed typical behavioral features of autism. In another study, mice developmentally exposed to high concentrations of diesel exhaust particles exhibited altered behavioral phenotypes including effects on locomotor activity and repetitive behaviors [15].

It has been suggested that airborne particulate matter may act like a Trojan horse [16] and represents an effective delivery system for diverse environmental toxicants to reach the brain. Additionally, associated water soluble compounds may provide a toxic stimulus independent of the particle composition itself and may be transported to the brain by the circulation system [17]. The toxicity of particulate matter in the lung have been linked to both the particulate constituents including metallic elements, oxidants, and oxidant forming species [18, 19] and the physical characteristic of particles itself [20]. Many compounds present in the particulate matter are neurotoxic [19]. For example, environmental exposure to neurotoxicants such as iron (Fe), copper (Cu), manganese (Mn), aluminum (Al), zinc (Zn), and lead (Pb) can induce oxidative stress [21, 22], and the brain is vulnerable to oxidative stress due to its great metabolic activity and low levels of antioxidants such as catalase (CAT) [23]. Previous studies have suggested that autism could result from the interaction between genetic and environmental factors with oxidative stress as the link between them [24]. Disturbing redox signaling, imbalance in the cellular redox state towards the pro-oxidant status, oxidative stress, and the resulting systemic inflammation are a possible mechanism of air pollution induced autism [25]. In addition, oxidative modification can modulate activity of several proteins that have relevant roles in normal brain function. Reactive oxygen species (ROS) play a crucial role in cell signaling. Oxidative stress also plays a role in controlling the activities of receptor proteins [26].

Extensive research has established the possible ability of the hypothalamic neuropeptide oxytocin (OXT) to modulate social behaviors across species including humans [27]. Results from the animal studies led to examination of the effects of OXT administration to humans and investigations of the etiology and treatment of psychiatric disorders, especially ASD [28]. Because social behavior symptoms are a clear manifestation of this disorder, the OXT system has been implicated in the biology of ASD, and has become a promising treatment option for the social symptoms of ASD. Increased research efforts into the potential involvement of OXT and its receptor (OXTR) in ASD include genetic studies, analysis of OXT levels in biological fluids and the assessments of OXT treatment in humans [29]. However, recent evidence investigating and implicating the role of OXTR protein in ASD has been increasing [29].

Epidemiological studies suggest an association between elevated air pollution and ASD. Since the effects of lower concentration of air pollution (that reflect the real world) on autism spectrum disorder is poorly understood. Hence we hypothesized exposure to ambient air pollution is a potential contributor to ASD. Previous studies have reported that simultaneous exposure to particulate matter and gaseous pollutants during pregnancy have been associated with ASD [30]. However, there is limited evidence showing a relationship between only gaseous pollutants exposure and ASD. Therefore, we investigated the effect of gaseous pollutant only exposures on ASD. Studies on rodents have found that prenatal exposure to VPA induces an animal model of ASD showing similar structural, functional, and behavioral features to human autistic patients [31–33]. Hence we compared the effects of the air pollution to that of VPA in rats. In the present study, we investigated the effects of prenatal and early life PM_2.5_ and gaseous pollutants (NO_2_, CO, O_3,_ SO_2_) exposure on a battery of behavioral dimensions, OXTR protein expression, and antioxidants (CAT) enzymatic activity and GSH concentration in the brain. Since the precise etiology of ASD remains poorly understood [34]., we assessed whether changes in OXTR levels may serve as part of the mechanism linking PM_2.5_/gaseous pollutants and ASD.

## Results

### The concentration of PM_2.5_ and gaseous pollutants

The average concentration of PM_2.5_ in the exposure period (E_0_ until PND_22_) was 43.82 ± 21.12 μg/m^3^. The average concentrations of CO, NO_2_, SO_2_ and O_3_ were 13.5 ± 2.5 ppm, 0.341 ± 0.100 ppm, 0.275 ± 0.07 ppm, and 0.135 ± 0.010 ppm, respectively. The concentrations of gases in both chambers one and two were equal. Control group of rats were exposed to clean air with these characteristics: PM_2.5_ < 5 μg/m^3^, SO_2_ < 0.02 ppm, NO_2_ < 0.04 ppm, CO < 2.4 ppm, and O_3_ < 0.02 ppm.

### The concentration of metals and PAHs

Concentrations of PM_2.5_-bounded heavy metals were determined, and the mean values can ordered as follow: Ca > Al > Na > Cu > Fe > Cd > Cr > Ni > Pb > Zn > Mn > As>V (Additional file 1: Table S1). The mean total concentration of 16 PAHs was 45.88 ± 21.02 ng/m^3^ (Additional file 1: Table S2). The order of average concentrations of the observed PAHs was phenanthrene> naphtalene> benzo(k)fluoranthene> florene> pyrene> anthracene> acenaphtylen> benzo(b)fluoranthene> chrysene> fluorantene> benzo(a) anthracene > acenaphten> dibenzo(a,h)anthracene> benzo (g,h,i)perylene> benzo(a)p- yrene> indeno (1,2,3-cd)pyrene.

### Open field

**Fig. 1:**
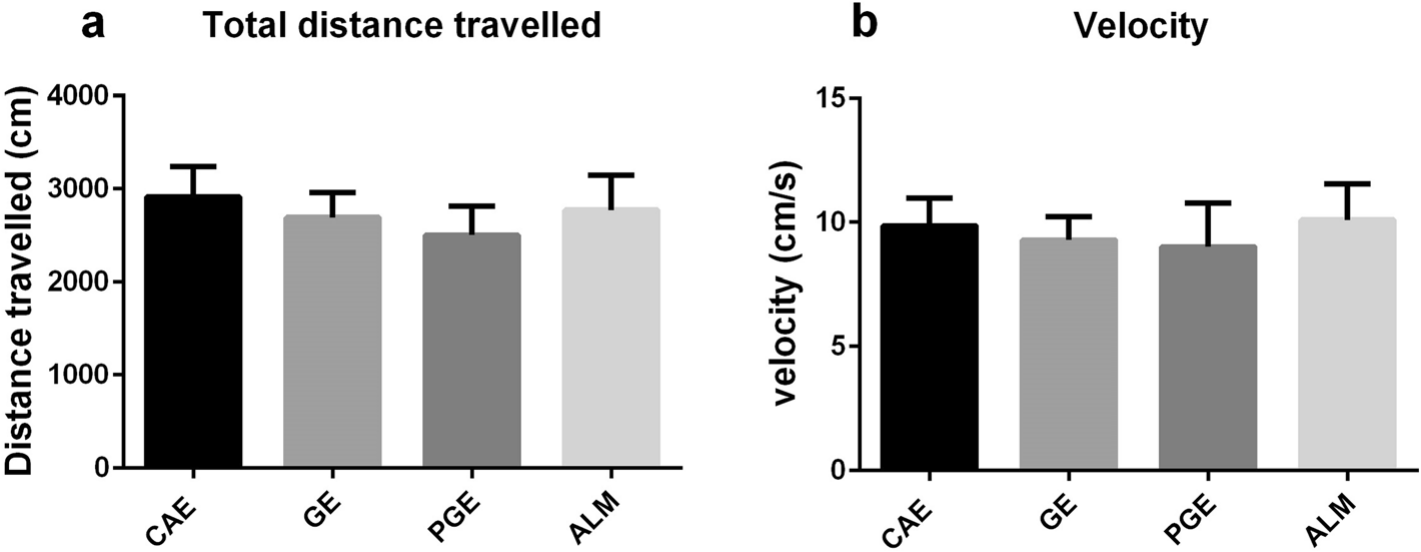
Open field test; PGE and GE rats showed no differences in locomotor behaviors. Prenatal exposure of PGE and GE rats did not affect: **a** distance travelled and **b** velocity in the open field task when compared to CAE control rats. (one-way ANOVA with Tukey ˀs multiple comparisons). Number of rats per group: CAE = 8, GE = 13, PGE = 8, ALM = 8

The open field test assesses locomotor activity and exploratory drive (23). To directly assess whether prenatal and early life exposures in the PGE and GE rats alter motor activity, the rats were assessed for locomotor activity (Fig. 1). There were no significant differences among the exposed rats and controls in total distance travelled and velocity.

### Social preference test

The main aim of this study was to assess whether prenatal and early life air pollution exposure could induce autistic traits in PGE and GE rats. To eliminate possible confounding, we set a control group (CAE rats) to distinguish between the effects of PM_2.5_ and gasses exposure (PGE rats) and gaseous exposure (GE rats) on the behavioral outcomes of pups. We also created an autism-like model by injecting doses of VPA to produce ALM rats, and compare these animals to the other exposure groups.

**Fig. 2:**
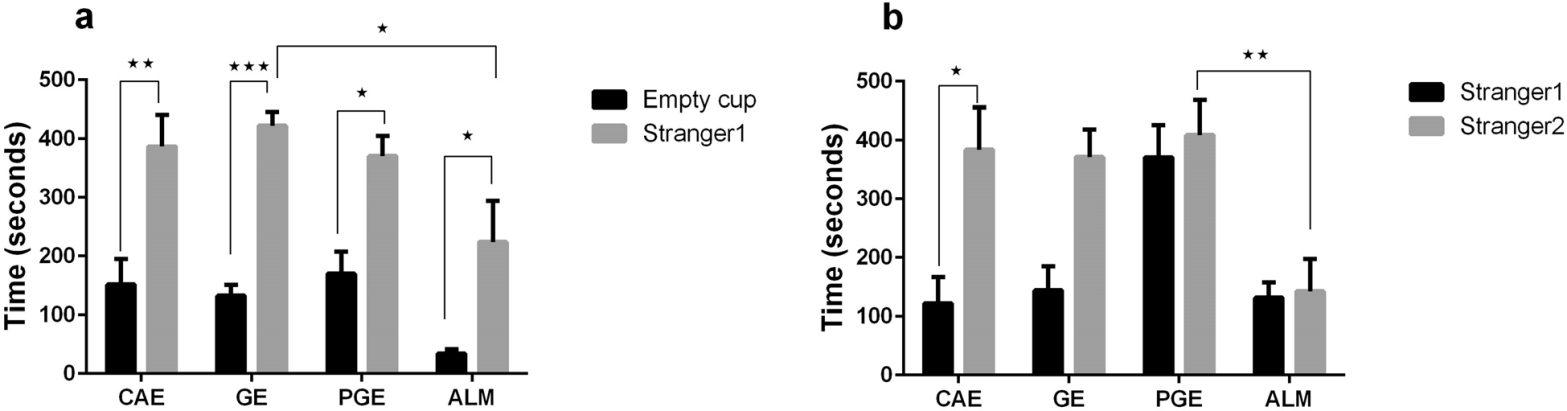
Three Chambered Social Preference Test. **a** In the sociability phase of the three chambered social preference test, sociability was assessed by measuring the cumulative time spent by the test rat in chamber containing the stranger1 vs. empty cup. There were no differences among the CAE, GE, PGE, and ALM rats in exhibiting preference toward stranger1over the empty setup (*p < 0.05, **p < 0.01, ***p < 0.001 Two-way ANOVA with Tukey ˀs test for the multiple comparisons). **b** In the social novelty test, the cumulative times spent by a test rat in the chamber containing stranger2 vs. stranger1 were compared. The error bars represent the standard error of the mean. (*p < 0.05, **p < 0.01 Two-way ANOVA with Tukey ˀs test for the multiple comparisons). Number of rats per group: CAE = 10, GE = 11, PGE = 9, ALM = 8

The first session of the social preference test allowed estimation of the social affiliation and motivation of subject rat. Typically, a wild type animal will spend a significant amount of time in the compartment with the stranger compared to the compartment with empty cup. Such behavior indicates normal sociability, social motivation, and affiliation [35, 36]. In the sociability session of the test, every group demonstrated a significant preference for spending time in the chamber containing stranger1 compared to the empty chamber. The time that the ALM rats spent in the chamber containing the stranger1 was significantly lower than that for the GE rats (Fig. 2a).

The second session of the test estimated social novelty and social memory. The ability to differentiate social novelty was determined by measuring time spent in the chamber containing stranger2 compared to the time spent in the chamber containing stranger1 that is now the familiar rat. The ALM and PGE rats showed no preference between the stranger2 and stranger1 rats. The time spent by ALM rats in the chamber containing stranger2 was significantly lower than that for the PGE rats (Fig. 2b).

### Y-maze

**Fig. 3:**
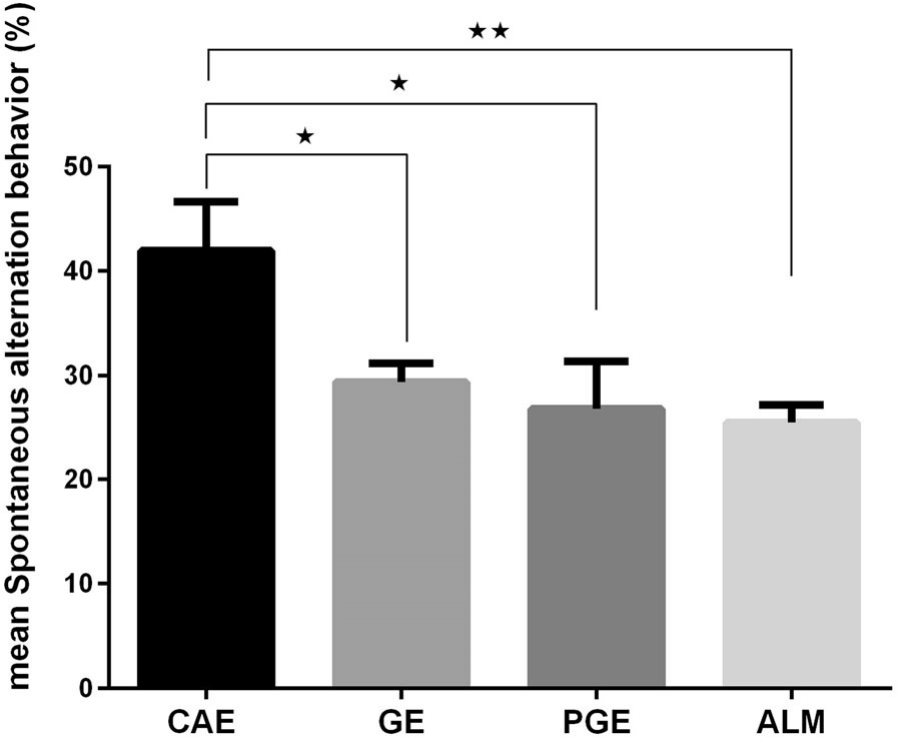
Y maze; a strong decrease in the percentage of alternation was detected in ALM, PGE, and GE rats. Error bars represent the SEM. (*p < 0.05, **p < 0.01 one-way ANOVA with Tukey ˀs multiple comparisons. Number of rats per group: CAE = 9, GE = 10, PGE = 8, ALM = 13

We assessed spontaneous alternation in the Y-maze to analyze respective behavior and /or working memory. Spontaneous alternation in a Y- maze represents a common exploratory strategy and repetitive behavior [33, 37]. The Y-maze labyrinth is a hippocampus dependent task of the spatial working memory. A decrease in the percentage of alternation in this test was detected in ALM, PGE, and GE rats, comparing to CAE rats (Fig. 3). The reduction in alternation in the Y-maze test also indicated restricted behavioral patterns (another core of ASD), in ALM, PGE, and GE rats [38].

### Marble burying

**Fig. 4:**
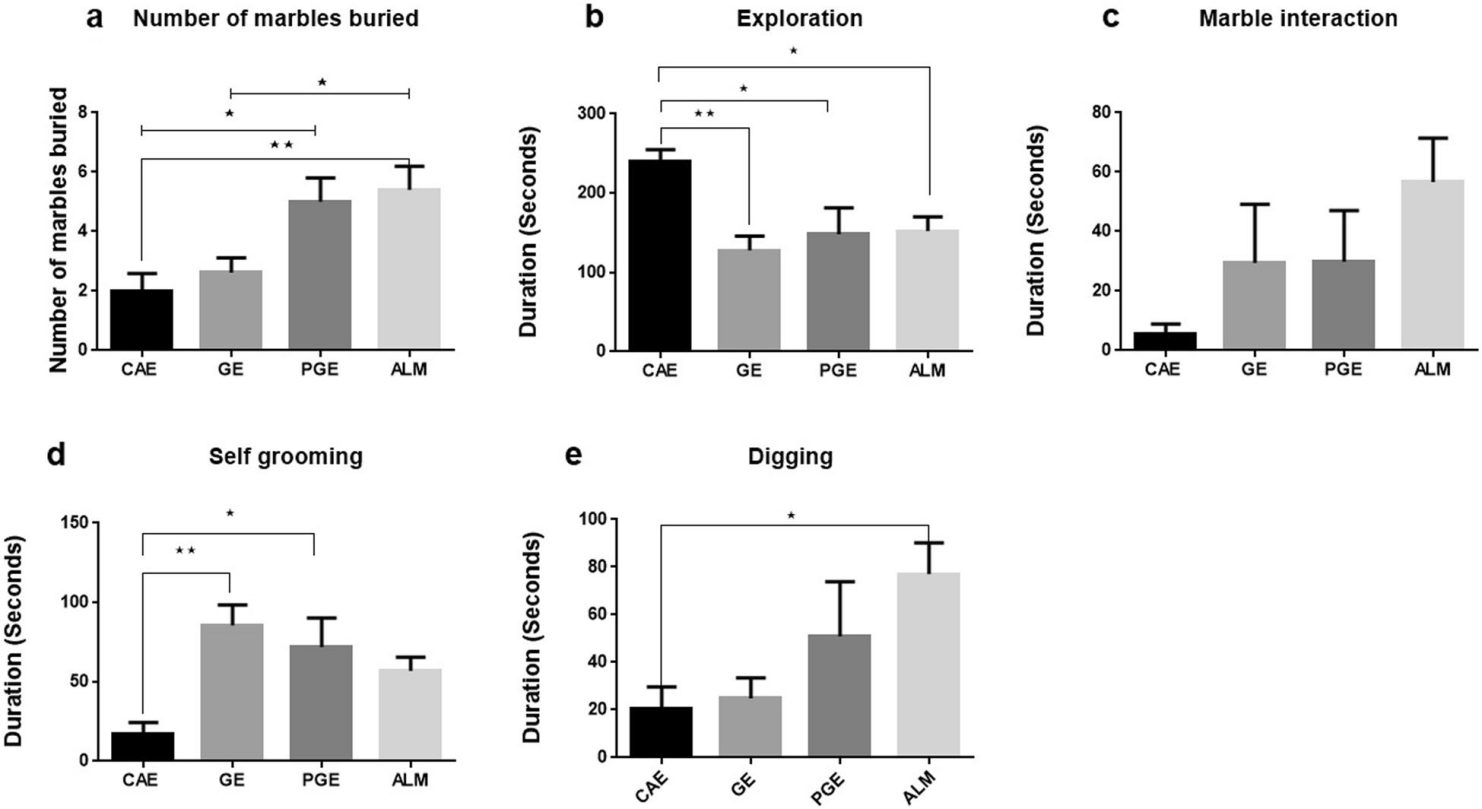
Marble burying test; **a** ALM and PGE rats buried more marbles vs CAE rats. However, GE rats buried less marbles than ALM rats. (*p < 0.05;* < 0.01one-way ANOVA with Tukey ˀs multiple comparisons) **b** GE,PGE and ALM rats spent less time in exploring in plexiglas test cage.(*p < 0.05;* < 0.01one-way ANOVA with Tukey ˀs multiple comparisons) **c** ALM,PGE and GE rats spent more time in interacting with marbles vs CAE group, but this increasing trend is not statistically significant.(one-way ANOVA with Tukey ˀs multiple comparisons) **d** GE and PGE rats spent more time in self-grooming as a repetitive behavior vs CAE group.(*p < 0.05;* < 0.01one-way ANOVA with Tukey ˀs multiple comparisons) **e** ALM rats spent more time in digging behavior vs CAE group,and also there is a trend of increasing digging behavior in PGE rats vs CAE group,but this is not statistically significant. Error bars represent the SEM. (*p < 0.05; one-way ANOVA with Tukey ˀs multiple comparisons). Number of rats per group: CAE = 8, GE = 8, PGE = 8, ALM = 10

Repetitive behavior was assessed by the marble burying test. The ALM and PGE rats buried more marbles than the CAE rats (Fig. 4a). For all the affected rats (ALM, PGE, and GE), the duration of exploration decreased vs CAE rats (Fig. 4b). For all the affected rats (ALM, PGE, and GE), there were increasing trends in the interaction time with the marbles compared to CAE rats although the difference was not significant (Fig. 4c). In this test, the frequency of self- grooming events and repetitive digging behavior were quantified. For the PGE and GE rats, the duration of self–grooming increased compared to the CAE rats (Fig. 4d) and the duration of digging in the ALM rats increased vs the CAE rats (Fig. 4e).

### Oxidative stress

**Fig. 5:**
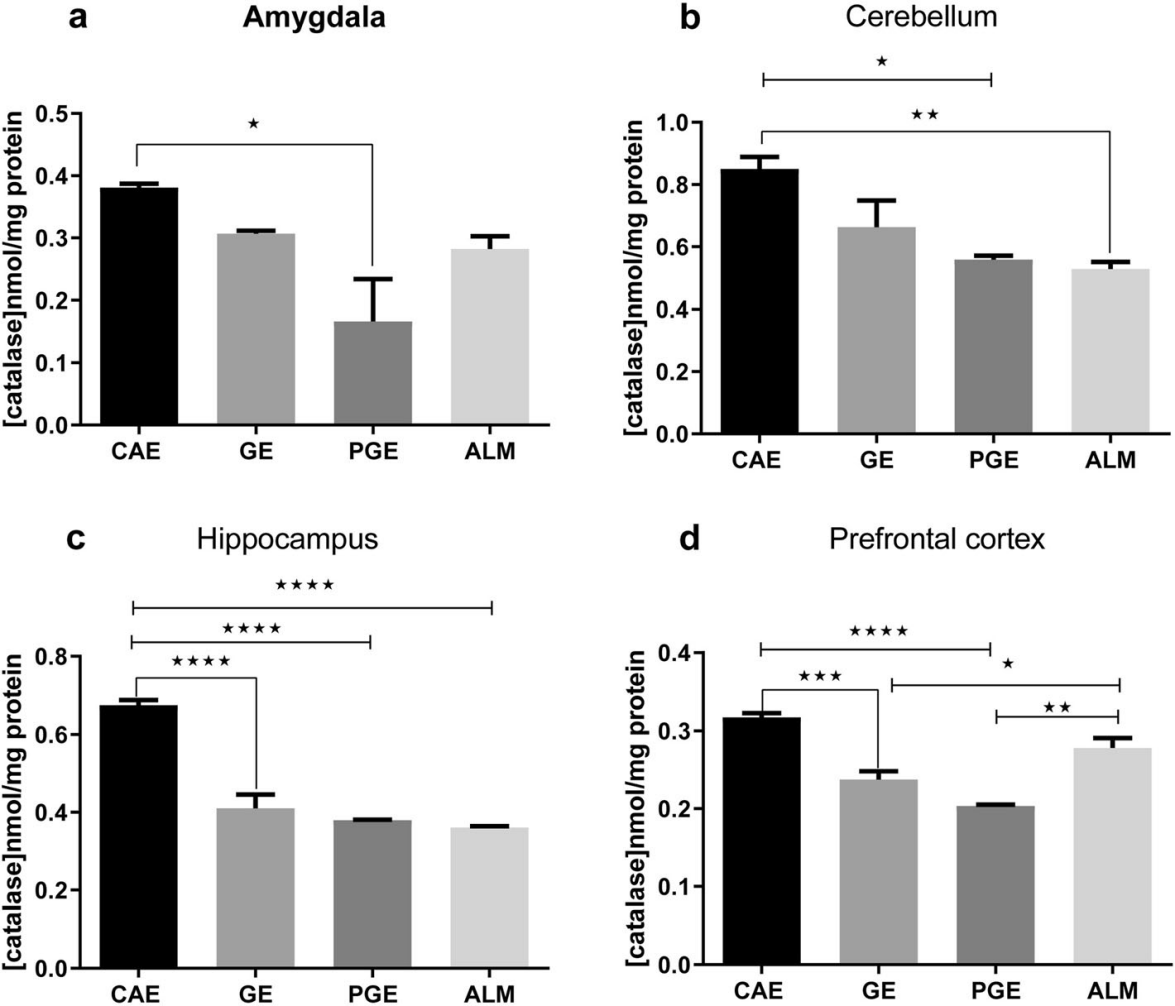
Catalase enzyme activity after prenatal and early life exposure of the CAE, GE, PGE, and ALM rats in the **a** amygdala (*N* = 3, Two times repetition), **b** cerebellum (*N* = 6, Three times repetition), **c** hippocampus (*N* = 6, Three times repetition), and **d** prefrontal cortex (*N* = 6, Three times repetition). The error bars represent the SEM. (*p < 0.05, **p < 0.01, ***p < 0.001, ****p < 0.0001; one-way ANOVA with Tukey ˀs multiple comparisons)

Oxidative stress was assessed by measuring the antioxidant enzymes i.e. catalase (CAT) and GSH in prefrontal cortex, amygdala, hippocampus, and cerebellum of the excised brain tissue. Overall, the CAT activity decreased significantly in all brain regions of the PGE rats that were tested compared to the CAE rats as a control (Fig. 5a). Although, the CAT activity in ALM rats did not significantly decrease vs CAE rats in amygdala and prefrontal cortex, this result may be due to an increased level of ROS-mediated cell damage, specifically ·OH that could result in an increase of CAT activity to maintain redox homoeostasis (Fig. 5. a, d) [25]. A significant decrease of CAT activity in the cerebellum was observed in the ALM and PGE rats compared to the CAE rats (Fig. 5b). The PGE, GE, and ALM rats exhibited significantly decreased CAT activity in hippocampus compared to the CAE rats (Fig. 5c). Thus, in the cerebellum, and amygdala, the activity of CAT activity was relatively similar to that in the PGE and GE rats vs CAE rats. The PGE and GE rats showed significantly decreased CAT activity in the prefrontal cortex compared to the CAE rats (Fig. 5d).

**Fig. 6:**
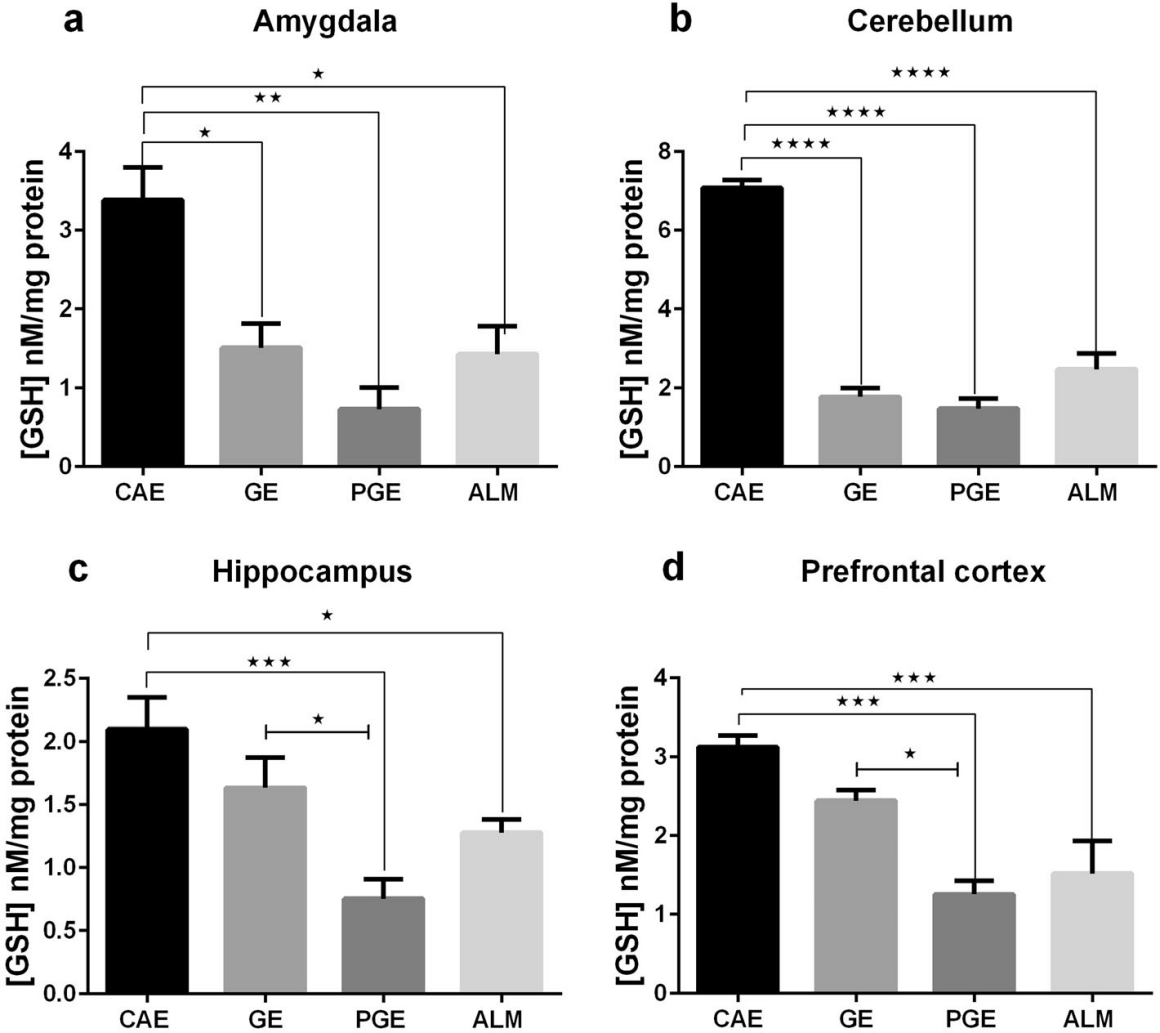
GSH levels after prenatal and early–life exposure of the CAE, GE, PGE, and ALM rats in the **a** amygdala (*N* = 3, Two times repetition), **b** cerebellum (*N* = 6, Three times repetition), **c** hippocampus (*N* = 6, Three times repetition), and **d** prefrontal cortex (*N* = 6, Three times repetition). The error bars represent the SEM. *p < 0.05, **p < 0.01, ***p < 0.001, ****p < 0.0001; one-way ANOVA with Tukey ˀs multiple comparisons)

As illustrated in Fig. 6, the PGE and ALM rats exhibited decreased levels of GSH in the prefrontal cortex, amygdala, hippocampus and cerebellum compared to the CAE group. The PGE, GE, and ALM rats showed significantly decreased levels of GSH in the amygdala and cerebellum (Fig. 6. a, b). Unlike the GE group, the ALM and PGE rats showed significantly decreased levels of GSH in the hippocampus and prefrontal cortex (Fig. 6. c, d).

### Expression of oxytocin receptor

**Fig. 7:**
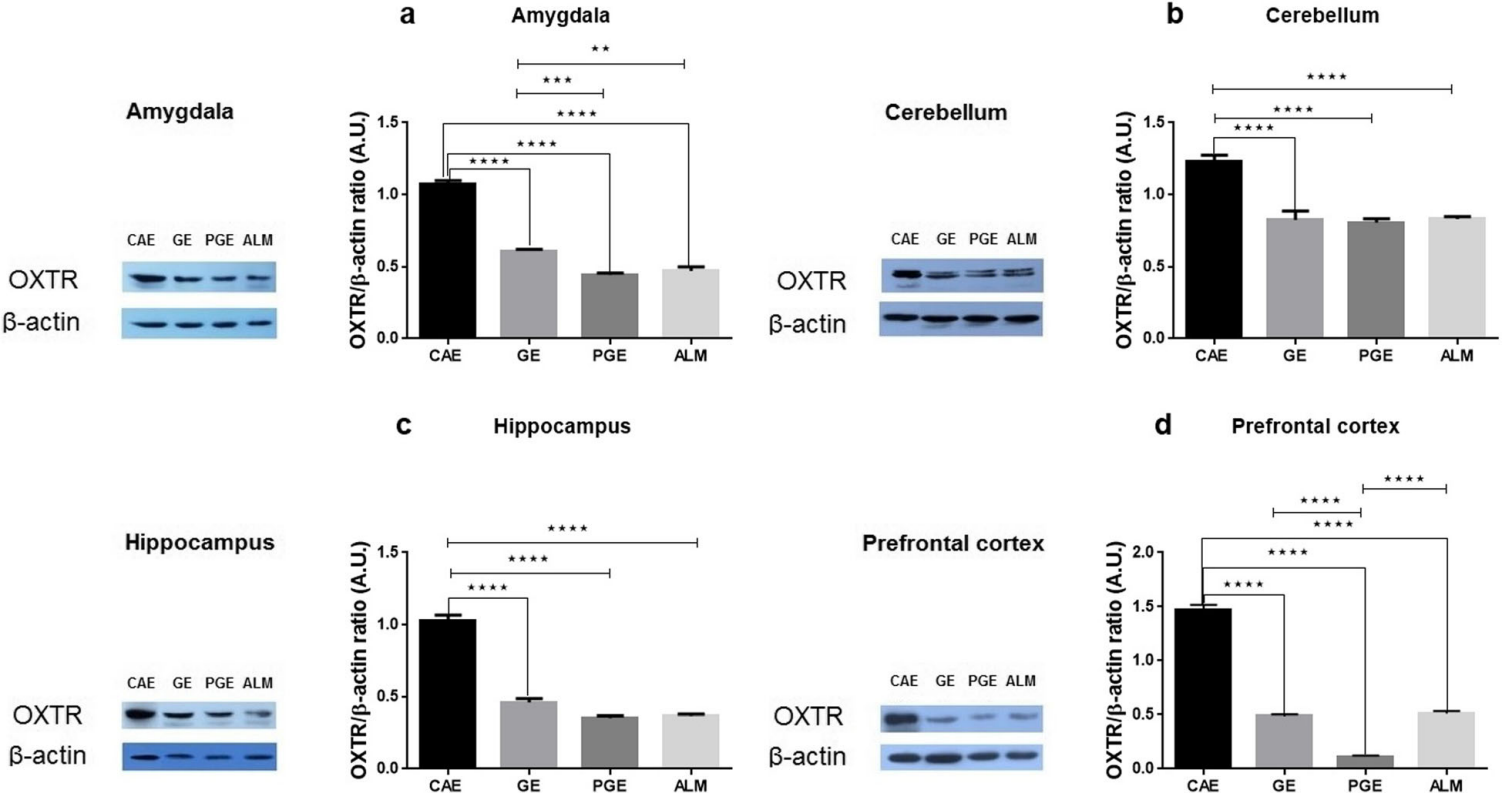
OXTR levels after prenatal and early–life exposure of the CAE, GE, PGE, and ALM rats in the **a** amygdala (*N* = 4, Three times repetition), **b** cerebellum (*N* = 4, Three times repetition), **c** hippocampus (*N* = 4, Three times repetition), and **d** prefrontal cortex (*N* = 4, Three times repetition). Error bars represent the SEM. *p < 0.05, **p < 0.01, ***p < 0.001, ****p < 0.0001; one-way ANOVA with Tukey ˀs multiple comparisons)

Western blot tests were conducted to determine the OXTR protein expression level. The ALM, PGE, and GE rats exhibited significantly decreased OXTR levels in the amygdala, cerebellum, hippocampus, and prefrontal cortex compared to the CAE rats (Fig. 7 a, b, c, d). These results indicate that the exposed rats showed the values similar to the autism-like model rats.

## Discussion

In this study, several groups of rats were exposed to ambient air pollution without any modifications during the period of E0 to PND21 covering the main neurodevelopmental events. As a result, the PGE, GE and ALM group of rats demonstrated decreased OXTR protein levels, reduced catalase activity and GSH concentration compared to the CAE group of rats (as a control group). However, the decrements in the GE rats were smaller than other symptomatic rats group. The PGE and GE rats showed similar results for these endpoints compared to the VPA rats. However, the GE rats showed smaller decrements in these measures.

The immature brains of fetuses or toddlers are more susceptible for environmental toxicants because the baby’s brain weight at birth is about 24% of its adult weight and the nerve cells are not fully developed until around the age of 2 years old. The developing nervous system is sensitive to environmental toxicants because temporal and regional developmental processes i.e., proliferation, migration, differentiation, synaptogenesis, myelination and apoptosis, grow during this period. During this vulnerable period, a wide range of chemicals may interfere with one or more of these processes through inhalation. The susceptible portion of the neurodevelopmental period in a rat are E0 to PND21 [12].

The immature rat’s vulnerability to environmental toxins may be related not only to the neurodevelopmental stages but also to the failure of other protective barriers e.g., the placental barrier and the blood brain barrier. The placental barrier should protect the fetus against the passage of the harmful substances like environmental toxins from the mother’s body. However, the placenta is not an effective protective barrier against environmental toxins during this time of extreme fetal vulnerability [39]. Although previous studies have described the associations between prenatal ambient air pollution exposure and impaired birth outcomes [40], it remains uncertain exactly what adverse effects are induced in the fetus. Various potential mechanism have been proposed including both direct particle translocation and/or through indirect mechanism such as intrauterine inflammation [41–43]. In recent years, studies were conducted showing that only nano-sized particles can pass the placental barrier [44, 45]. However, a recent study [46] found that black carbon particles were able to translocate from the mother’s lungs to the placenta. An indirect mechanism may also be involved since the exposure to particulate air pollution and its constituents e.g. PAHs and metals, can induce oxidative stress and inflammation that leads to developmental toxicity and adverse health outcomes [47, 48] by negatively affecting placental transport [49].

The aim of this study was to use ambient air pollution concentrations without any modification to mimic real world exposure scenarios. Since prior studies typically used high exposures, it is not clear that exposure at commonly observed air pollution concentrations can induce ASD or not. Comparing the internal dose of PM_2.5_ per kg body weight indicates that rat’s dose in our study is about 2–3 times higher than the equivalent human dose. However, these results rely on more realistic exposures compared to prior studies.

In the present study, the concentrations of PM_2.5_ and gaseous pollutants, along with the concentration of heavy metals and PAHs bounded to the PM_2.5_ were determined. A recent study conducted by Zarandi et al. [50] in Tehran found higher concentrations of metals than our study, possibly due to the different sampling periods. Lead, zinc, manganese, benzo(a) anthracene, benzo(k) fluoranthene, benzo(a) pyrene, and pyrene in PM_2.5_ particles may have neurotoxic potential, because these toxicants have been associated with the production of ROS [51–53]. While the PM_2.5_ and components bound to it are likely candidate for inducing autism-like phenotype, it is possible that gaseous pollutants particularly nitrogen oxides contribute to induce ASD [14, 54]. Since the diagnosis of ASD is based mainly on behavioral phenotypes, behavioral assessments based on the clinical symptoms in humans were used to detect ASD in rodent models [55, 56]. Therefore, we performed behavioral tests to support the important characteristics of the ASD i.e. deficit in social interaction and increase in restricted/repetitive behaviors.

To assess the sociability domain, a three chamber social experiment was conducted. Exposure to PM_2.5_ and gaseous pollutant did not affect sociability in social preference test. However, during the social novelty phase, the ALM and PGE rats showed no preference when presented to the novel and familiar rats, suggesting the inability to differentiate social novelty that has been reported to be an autism-like behavior [14, 57]. Our findings in this test were also in agreement with the studies of Li et al. [9] and Church et al. [58] that showed an inability to differentiate social novelty.

To assess the spontaneous working memory and repetitive behavior, the Y-maze test was conducted. The results of Y-maze test showed decreased percentages of spontaneous alternation behaviors and consequently memory defects in the PGE, GE, and ALM rats compared to the CAE rats. The reduction of alternation in the Y-maze test could also indicate a restricted behavioral pattern [38].

The marble burying test was used to assess repetitive behaviors in rodent models of autism as well as in models of obsessive-compulsive behavior. In the marble burying test, evenly spaced marbles served as a proxy for repetitive digging behavior. Increased repetitive digging results in more marbles being buried. Our results showed that the number of marbles buried and the digging time of the PGE rats was greater than those in the CAE rats, suggesting increased repetitive behaviors [33]. In contrast to our observations, PM_2.5_-exposed rats in another study barely buried the marbles, indicating no increase in repetitive behaviors [9].

The open field test showed that there were no differences between the PGE, GE, and even the ALM rats with the control rats (CAE) in motor activity. Our findings are in agreement with two recent studies by Chang et al. [14] and Church et al. [58]. Previous studies had suggested that the oxidative stress due to the biological enzyme activity caused by exposure to PM_2.5_ drives such behavioral changes [59].

The role of oxidative stress in inducing autism may be through an imbalance between the generation of ROS and the defense mechanisms against ROS by antioxidants. GSH plays an important role in several cellular process such as apoptosis, and is also involved in the anti-ROS defense system. GSH is the most important antioxidant for detoxification and elimination of environmental toxins [60]. Therefore, GSH destruction can increase susceptibility to oxidative stress. GSH acts as a proton donor, neutralizes H_2_O_2_, reacts directly with radical species, and also contributes to regeneration of other antioxidants [61]. Catalase protects cells from oxidative stress by catalyzing the rapid decomposition of H_2_O_2_ through two types of reactions depending on its peroxidation and catalytic activities [62]. CAT is also involved in the recycling of the cellular H_2_O_2_ [25].

Our findings showed decreased levels of GSH and CAT in the exposed groups of rats compared to the control rats. This result is similar to the observations by James et al. [63] and by Zoroglu et al. [64] that showed reduced levels of GSH and CAT in autistic patients, respectively.

Due to the complexity of symptoms seen in autism, different areas of the brain are likely to be involved in ASD. Among them, four important areas including hippocampus, amygdala, prefrontal cortex, and cerebellum play important roles in the neuropathological changes. The amygdala affects social information and emotional interpretation as well as fear and anxiety responses [65, 66]. Several postmortem morphometric studies have documented developmental alterations in the cerebellum related to ASD [67]. Neocortical studies on autism have found a 67% increase in the number of neurons in prefrontal cortex [68]. Similarly, a connection has been reported between ASD and neuronal size abnormalities in medial temporal lobe structures including the hippocampus [69, 70].

One of the main objectives of this study was to assess the effect of exposure to the mixture of PM_2.5_ and gases (PGE) and gases alone (GE) on the levels of the oxytocin receptor protein (OXTR). Some associations have been previously observed between OXTR haplotypes, ASD, IQ, and total VABS scores in humans [71]. In the present study, decreased levels of OXTR in the prefrontal cortex, amygdala, hippocampus, and cerebellum were observed in the exposed and ALM rats compared to the control rats. These results are similar to those of Bertelsen et al. [72] in which the level of OXTR decreased in the autistic rat model induced by VPA. The possible mechanism for down-regulation of OXTR protein at nerve terminals was assumed to be the increased oxysterols levels during neuronal injury [73]. There are no prior reports of the role of OXT and OXTR in ROS production in the nervous system. Thus, further studies are required to establish this relationship. Denda et al. [74] found that OXT is expressed in human skin keratinocytes and released in response to a calcium influx via P2X receptors. Also, there are evidence that OXT is not only expressed in keratinocytes, but also in human skin-derived dermal fibroblasts [75], which are in common with neurons in ectodermal derivation. In addition, the role of OXTR expression in fibroblast production has been reported [76]. Expression of OXT occurs in all epidermal layers, while expression of the OXTR only occurs in the basal layers [75].

There is evidence that with inhibition of OXTR signaling in integumentary system, OXT exerts its effects through the alteration of oxidative stress, intracellular GSH levels, and cytokine release by dermal fibroblasts and keratinocytes. Increments of susceptibility to oxidative stress occur following the reduction of OXTR in dermal cells [75]. Simultaneous to increment of ROS levels in OXTR reduction in dermal fibroblasts and keratinocytes, a decrement of intracellular GSH concentrations has been reported [75]. Some other studies have also shown the role of OXT and OXTR in oxidative stress and GSH levels. For instance, sepsis-induced pelvic inflammation caused by the increase of ROS and reduction of GSH levels, could be treated with OXT administration [77] or with atosiban, an OXTR antagonist that increased oxidative stress in the cardiomyocytes of the newborn rats whose mothers received atosiban during gestation [78]. Previous studies revealed that OXTR is associated with social cognition [79–81] in autism [82]. The loss of oxytocin or OXTR may result in reduced social recognition [83] and social interaction [84].

In recent years, several studies have shown the association between exposure to particulate matter and increased risk of ASD [85, 86]. In addition, the relationship between ambient particulate matter and autism-like phenotype has been recently assessed in rodent models. In a study by Li et al. [9], Sprague-Dawley rats were exposed to PM_2.5_ by intranasal instillation. However, Li et al. [9] suggested that the whole-body inhalation exposure (such as the current study) is more physiologically relevant to human context rather than intranasal instillation. In addition, the dose to the rat pups in the Li et al. (2018) study was determined using the adult rat’s respiratory rate. Thus, this dose was higher than typical exposures in humans. In the current study, rats were exposed to the concentrations of PM_2.5_ and gaseous pollutants at ambient levels. Therefore, this model of exposure may better reflect the actual human exposure to PM_2.5_ and gaseous pollutants.

## Conclusions

In the present study, our results showed that prenatal and early life exposure to a mixture of PM_2.5_ and gaseous pollutants (PGE rats) and gaseous pollutants alone (GE rats) caused behavioral deficits, including increased repetitive behavior, poor social interaction and an inability to differentiate social novelty. Air pollution also affected oxidative stress biomarkers like CAT and GSH, and may induce down-regulation of OXTR protein in male rats. To our knowledge, this is the first report that compares the effects of exposure to PM_2.5_ and gaseous pollutants (PGE rats) and gaseous pollutants alone (GE rats) with VPA-induced rat models of ASD (ALM rats). These experimental findings support the hypothesis that an etiological association exists between a complex mixture of air pollution and physiopathology of ASD. Further mechanistic research should be performed to determine the mode of neurodevelopmental toxicity of air pollution on ASD.

## Methods

### Rats

Wistar rat litters of both sexes were purchased from Pasteur Institute (Tehran, Iran). The rats were housed at the Shahid Beheshti University of Medical Sciences under specific pathogen free (SPF) and standard conditions, including access to supplies of water and food ad libitum under a 12 h light/dark cycle. The rats were maintained under constant environmental conditions including temperature of 20–25 °C and relative humidity of 40–60%. The ethical use of animal models was approved by Shahid Beheshti University of Medical Sciences’ Ethics Committee.

### Study location and method of exposure

**Fig. 8:**
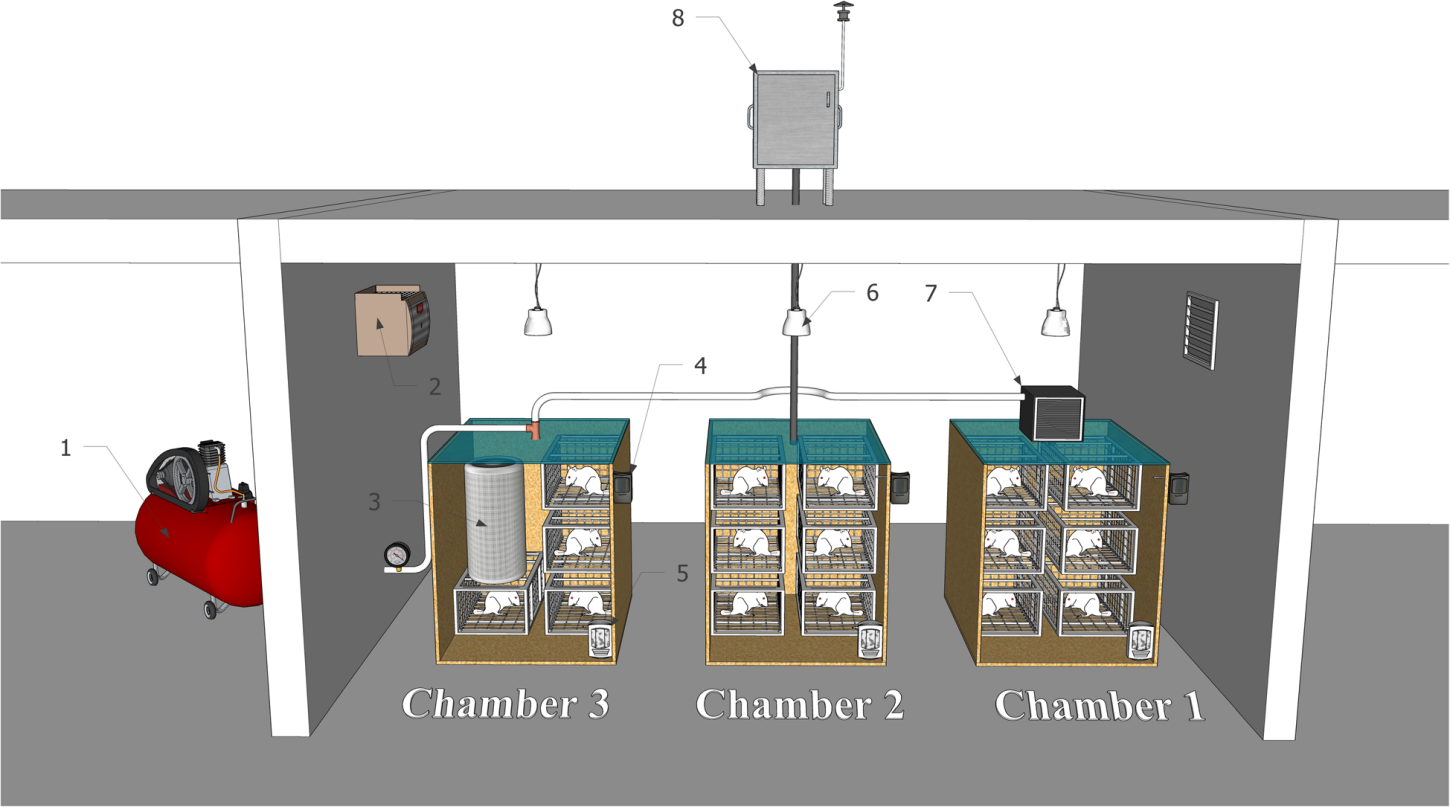
Schematic of exposure method: 1- Oil-free compressor, 2- Thermometer, 3- Air purifier, 4- Moisture Sensor, 5- Dust Track, 6- Time lamp, 7- HEPA Filter, and 8- Echo PM

The pilot animal (Fig. 8) room was located on the roof of the School of Public Health and Safety of the Shahid Beheshti University of Medical Sciences (35.7991 ° N, 51.3947 ° E) at a height of 20 m (fourth floor) above the ground.

Four groups of rats including PGE (particulate matter and gaseous pollutants), GE (gaseous pollutants), CAE (clean air) and ALM (autism like model exposed to clean air) rats were exposed in the animal pilot study room. The GE rats in chamber 1 were exposed to filtered ambient air at a flow rate of 20 L/min provided by an oil-free compressor and a HEPA filter (model H13) to remove particulate matter. PGE rats in chamber 2 were exposed to ambient air at a flow rate of 20 L/min using an Echo PM_2.5_ Low Volume Sampler (LVS) (TCR Tecora Italy) without a filter to provide exposure to both PM_2.5_ and gaseous pollutants. The CAE and ALM rats were housed in chamber 3, and exposed to cleaned ambient air at 20 L/min flow rate provided by an oil-free compressor and two purifier systems (Model: Air Touch A5, Honeywell, and model: KAIST-AIR Home) to remove both particulate and gaseous pollutants. Pregnant Wistar rats in the ALM group received Valproic Acid (VPA: 350 mg/kg) at gestational day 12.5 to induce ASD.

### Drug administration

Before the start of the experiment, the rats at the age of 10 weeks were acclimated to the pilot animal room for 1 week. The rats were then mated in-house at the age of 11 to 13 weeks, and pregnancy was determined by a vaginal plug on embryonic day 1 (E1).

**Fig. 9:**
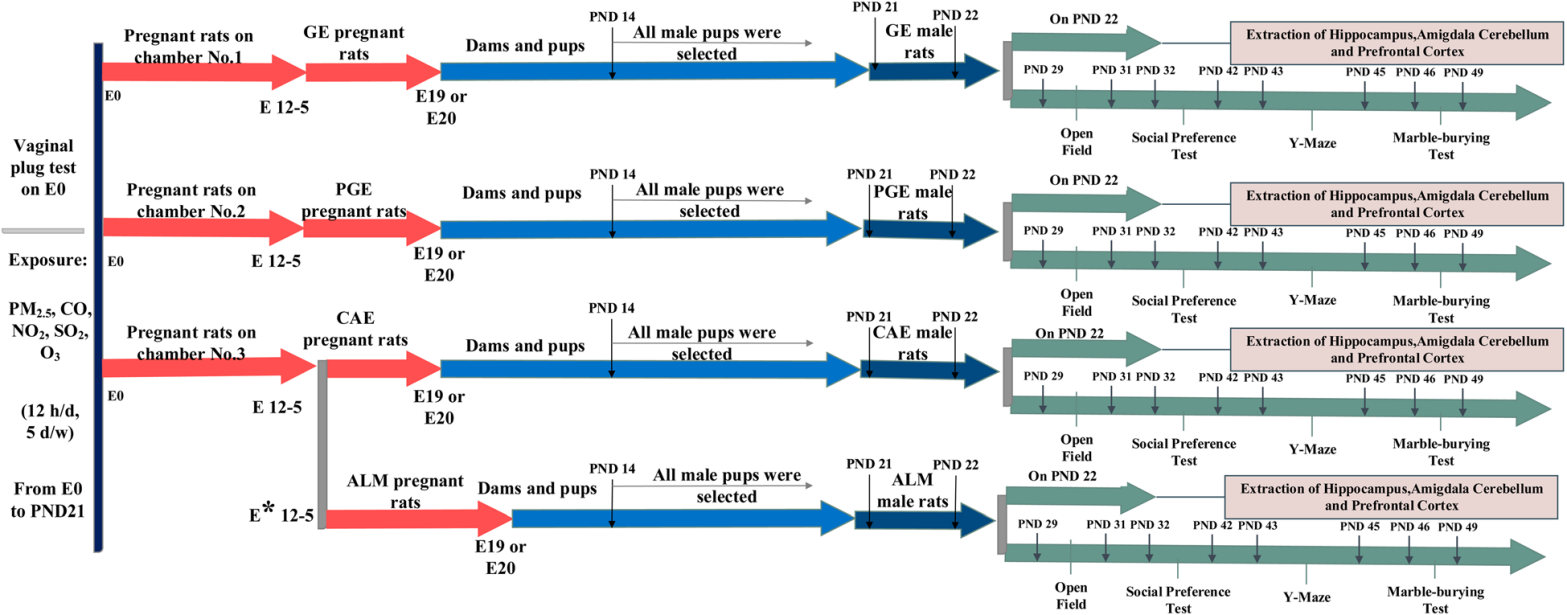
Experimental design of study: rats were time mated and exposed to PM_2.5_/gases and gases alone from E0 to PND21. Biochemical tests were started at PND22 by extraction of brain tissue. Behavioral assessments were started at PND 29. * VPA-dams received a single intraperitoneal (i.p.) injection of VPA at a dose of 350 mg/kg body weight

To produce the ALM rats [87], the sodium salt of valproic acid (P4543 - Sigma) was prepared in 0.9% saline solution (100 mg/ml, PH 7.3). On E12.5, when the pregnant rat’s weight was 200–225 g, VPA-dams received a single intraperitoneal (i.p.) injection of VPA at a dose of 350 mg/kg body weight [88]. Control dams received a single injection of saline solution (i.p., 0.9%) (Fig. 9).

### Exposure periods

Pregnant dams and pups were exposed to ambient air that contained PM_2.5_, CO, NO_2_, SO_2_, and O_3_ for 12 h per day and 5 days per week (Saturday to Wednesday) from embryonic day 0 (E0) to postnatal day 21 (PND21). The concentrations of PM_2.5_ and gaseous pollutants (CO, NO_2_, SO_2_ and O_3_) in each chamber were measured using a DustTrak model 8520 and an AeroQual 500, respectively.

The rats in control groups were exposed to clean air during the same period. The exposure period was chosen based on the human epidemiological studies suggesting that air pollution exposure during all the three trimesters of pregnancy and the first 9 months of an infant’s life is related to ASD risk [30, 89, 90]. The exposure time was designed to cover the main neurodevelopmental events (including: the timing of neurogenesis, synaptogenesis, gliogenesis, oligodendrocyte maturation) that were happening in this window of susceptibility, the period of E0 to PND21 [91]. For the exposure of pregnant dams and pups, female rats (11–13 week old) were time mated with males (3 females with 1 male per cage) on Friday evenings. The copulatory plug was checked the next morning before the Saturday onset of the weekly exposure. Rats with confirmed vaginal plugs were removed from breeding cage on embryonic day zero and housed individually in cages in either chambers one, two or three. Chambers 1 and 2 housed nine pregnant dams; while chamber 3 housed eighteen dams for duration of their pregnancy, parturition, and weaning of the litters. The use of Friday timed mattings ensured that E0 occurred on a Saturday and ensured that the exposure timing was the same for all rats. All dams were exposed on the same gestational days E0–4, E7–11, E14–18 and all pups were exposed on PND1-PND7, PND10–14, PND17-PND21. Exposure stopped in chambers one and two while rats were giving birth on E19 and E20. On PND14, male and female pups were separated and female pups were removed from their mothers. The male pups remained with their mothers until weaning occurred on PND 21. On PND 22, the pups were divided into two groups. One group were euthanized by decapitation for the biochemical tests and the second group were transferred to housing racks (two rats per cage) 1 week before the starting the behavioral tests (on PND29) and for the duration of the behavioral testing period. Behavioral tests only were performed on male rat offspring and all behavioral tests were performed between 9:00 and 15:00. The behavioral assessments were made sequentially on a single cohort of male rats. This lack of replication is a limitation of this study (Fig. 9).

### Determination of PM_2.5_ characteristics

PM_2.5_ was sampled continuously during the exposure hours using an Echo PM Low Volume Sampler near the air intake for the exposure chambers [92]. PM_2.5_ was collected on 47 mm quartz filters at a flow rate of 20 L/min. Before sampling, the filters were washed with double-distilled water, and placed in an oven at 100–105 °C for 2 h [93]. After sampling, the filters were stored in aluminum foil at − 10 °C to prevent evaporation and photo-degradation of PM components. For each 2 days, a new filter was used for sampling. Also, field blanks were used to control for the possible contamination during the sampling procedures. For this, a blank filter was treated with the same manner as a filter used for sampling (placed into the sampler, placed into the aluminum foil, etc.), except that the sampler was not operated.

To determine the elemental composition of PM, one-half of filters were shredded, and put into a Teflon container with 2.5 ml of concentrated of HClO_4_ (70%) and 2.5 ml HNO_3_ (69%). Samples were heated at 170 °C for 4 h, and dried on a hot plate at 100 °C. After adding 2.5 ml of double-distilled water and 2.5 ml of HNO_3_,the samples were shaken at 180 rpm for 30 min [93]. Finally, the samples were filtered through Whatman No. 42 filters and diluted with double-distilled water to 10 ml. The samples were stored in plastic vials at − 4 °C until the analysis by ICP/MS (Agilent, Model: 7900) [94]. Laboratory blanks were used to control for the effects of laboratory contaminations. The metal concentrations are provided in the Additional file 1: Table S1. The concentrations of metals were analyzed in triplicate, and the averaged values were used. Field and laboratory blanks were used to control for the effects of field and laboratory contaminations. The limit of detection (LOD) and the limit of quantitation (LOQ), of Pb was 5 and 1 μg/L, respectively. The LOD and LOQ for other elements were 25 and 5 μg/L, respectively.

To determine the concentrations of polycyclic aromatic hydrocarbons (PAHs), the other section of the filter was placed in a Teflon container, and extracted with 2.5 ml of dichloromethane (CH_2_CL_2_) and 2.5 ml of methanol (CH_3_OH) using an ultrasonic bath at 20 kHz for 30 min (Elmasonic S 80 H). The extracts were filtered with 0.22 μm Millipore filters (Hesperia CA, USA). PAHs concentrations were determined using GC/MS (Agilent, model: 5890 A). The PAH results are provided in Additional file 1: Table S2. The concentrations of PAHs were analyzed in triplicate, and the mean concentrations were reported. In the analysis of all of the blank samples, PAHs were not observed. The values of LOD and LOQ for 16 PAHs were < 2 and < 10 ng/L, respectively.

### Behavioral tests

#### Open field

The open field test was conducted as previously described by Chang et al. [14] with some modifications. At PND29 for the male rat offspring, each rat was placed in a clean acrylic cage (60*60*60) with no bedding for 30 min. To assess general locomotor activity levels and willingness to explore in a novel environment, the rat’s location during the test was tracked using Ehtovision XT 7 system from the video recorded using a Microsoft LifeCam HD-6000.

### Social preference test

This test was conducted as previously described by Li et al. [9] with modifications. The three sessions in this test was conducted in a three chambered box (60*40*22 cm) equipped with retractable doorways that permitted access to each chamber.

This test was conducted at PND32 for each male rat offspring. In session 1 (habituation), a pup was placed in the middle chamber with the doorways open, and allowed to explore the other two side chambers. For session 2 (sociability), at the end of the period of habituation, pups were made to interact with a never-before-met and age-matched rat enclosed in a wire cup placed in a side chamber. An empty wire cup was placed in the other side chamber. During session 3 (social novelty), a new and unfamiliar rat was placed in the wire cup that had been empty during the previous session. Each session lasted 10 min, and the time spent in each chamber was manually recorded.

### Y-maze

The test was conducted as previously described by Grabrucker et al. [38]. Spontaneous alternation behavior was assessed at PND43 for each male rat offspring in a symmetrical Y Maze (3 arms, 40*9 cm with 16 cm wall height). Arm choices by the rats (all four paws entering one arm) were recorded while the rats were allowed to explore the Y-shaped labyrinth for 5 min. Alternation was determined by recording the order of the visited arms (A, B, or C). Overlapping triplets of three arm visits were counted as one complete spontaneous alternation. The percentage of alternation was calculated using the equation:

1 $$ Percentage\ of\ alteration=\frac{\mathrm{number}\ \mathrm{of}\ \mathrm{spontaneous}\ \mathrm{alternation}}{\mathrm{total}\ \mathrm{number}\ \mathrm{of}\ \mathrm{arm}\ \mathrm{visits}-2}\times 100 $$

where an alternation was recorded as consecutively visiting the three arms.

### Marble burying

This test was conducted as previously described by Ku et al. [95] with some modifications. On PND46 for each male rat offspring, the subject was placed in a standard Plexiglas test cage (42*24*17 cm) with a 5 cm deep layer of corncob bedding, and allowed to explore freely for 10 min. Each subject was placed in a transfer cage and 18 marbles (1.3 cm diameter, red) were placed on the bedding surface in a 3*6 pattern. The subject was then placed in the test cage, and allowed to explore for 10 min. After ten minutes, the subject was removed from the test cage, and the number of buried marbles was counted. A marble was considered as buried only if at least two-third of the marble was covered by the bedding material.

### Tissue collection

Studies have increasingly recognized that developing brains are more sensitive to both neuronal apoptosis that are likely to be affected by age-related injury vulnerability [96–101]. Since approximately 90% of the rat cortex is formed by PND 20 and the weight of rat cortex reaches approximately to 90% of its weight by PND 20, the typical age of weaning. Also, myelination peaks at approximately PND 20, when maturation markers especially myelin basic protein are detectable [102]. Thus, the biochemical assessments should be performed after the rats attain PND20 to ensure valid test results. Since exposures continued until PND 21, the following day (PND22) was selected for conducting the biochemical tests.

On PND22, sixteen male offspring in each group were anesthetized via CO_2_ and rapidly decapitated. The prefrontal cortex, amygdala, cerebellum, and hippocampus were rapidly dissected out, and frozen in liquid nitrogen at − 80 °C until use.

### GSH

The test was conducted as described by Ellman et al. [103]. Sixty μg of protein supernatant containing 19.8 mg of DTNB (D8130-1G SIGMA) in 100 ml, 0.1% sodium nitrate and phosphate buffer (PH 7.4) were used for the GSH assay. The absorbance was measured at 412 nm by an ELISA reader. Ellman’s colorimetric method measures the formation of GS-TNB complex from DTNB (5,5̉ dithiobis (2- nitro benzoic acid)) in which its reduction caused the development of a yellow color.

### Catalase

To determine the level of catalase activity, the concentrations of the yellow stable complex of ammonium molybdate and hydrogen peroxide were measured at 405 nm with an ELISA reader [104]. First, 20 μL of the sample was loaded in each well of the 96-well microplate. Then, 100 μL hydrogen peroxide (65 mM) was added to each well. This mixture was incubated for 4 min at 25 °C. Then, 100 μl of ammonium molybdate (32.4 mM) was added to each well, and the absorbance was read at 405 nm.

### Western blot

Western blotting was used to assess the level of OXTR in the prefrontal cortex, amygdala, hippocampus, and cerebellum of the male rats. On PND 22, the prefrontal cortex, amygdala, hippocampus, and cerebellum were excised. Next, they were lysed on ice via lysis buffer [50 mM Tris-HCl (PH 8), 0.25% sodium deoxycholate, 0.1% sodium dodecyl sulfate (SDS), 150 mM NaCl, 1 mM EDTA, 0.1%Triton X-100, complete protease inhibitor cocktail, phosphatase inhibitors cocktail] for 2 min. Then, the lysates were cleared with centrifugation at 16,100 Xg for 10 min at 4 °C. In order to determine the protein concentration, Bradford’s method [105] using bovine serum albumin (BSA) as standard was performed to denature proteins, equal volumes of 5X sample buffer were added to lysate proteins. Then 60 μg of total proteins were loaded into SDS-polyacrylamide gel electrophoresis and then transferred onto polyvinylidene difluoride membranes. Then blots were blocked by blocking solution [2% non-fat dry milk in tris-buffered saline Tween 20 (TBST) (containing 0.2% Tween 20, 50 mM Tris–HCl pH 7.4, 150 mM NaCl] for one hour and half. At this point, the membranes were incubated with the primary antibody (Anti-Oxytocin Receptor antibody [EPR12789]-20 °C) (Abcam ab181077-100 μl) at 4 °C overnight. Next day, the blots were washed with TBST three times for 10 min each. After incubating with the anti-rabbit horseradish peroxidase secondary antibody (Anti-rabbit IgG, HRP-linked Antibody) (cellsignaling) for 90 min at the room temperature, immunoreactivity was detected with ECL kit and captured by Kodak x-ray films. To compensate for loading errors, membranes were stripped with stripping buffer [100 mM 2-mercaptoethanol, 2% (w/v) SDS, 62.5 mM Tris-HCl (PH 7)] followed by incubating with anti β-actin antibody. Densitometric data of protein bands were obtained with ImageJ 1.41o.

### Statistical analysis

All data were analyzed by using Graph Pad software (Graph Pad Prism v6.0). For the three-chambered social preference test, time spent in the ‘stranger1’ versus ‘empty’ chambers (for the sociability phase) or ‘stranger1’ versus ‘stranger2’ chambers (for the social novelty phase) were compared with a two-way analysis of variance (ANOVA) followed by Tukey ˀs test for the multiple comparisons. A one-way ANOVA followed by Tukey ˀs test was used for the analysis of the other test results. Multiple comparison corrections were not done. The significance levels of 0.05, 0.01, 0.001and 0.0001 were applied in all analyses.

## Supplementary information

**Additional file 1: Table S1.** Concentration of Metals bound PM_2.5_ in exposure period. **Table S2.** Concentration of 16-PAHs bound PM_2.5_ in exposure period.

## Data Availability

The datasets used and/or analyzed during the current study are available from the corresponding author on reasonable request.
