# Supplementary material for: Effects of PM2.5 and gases exposure during prenatal and early-life on autism–like phenotypes in male rat offspring
Source: Part Fibre Toxicol. 2020 Jan 29;17:8. doi: 10.1186/s12989-020-0336-y (PMC6990481; doi:10.1186/s12989-020-0336-y)
Supplement: Supplementary file 1 — Additional file 1: Table S1. Concentration of Metals bound PM2.5 in exposure period. Table S2. Concentration of 16-PAHs bound PM2.5 in exposure period. [file 12989_2020_336_MOESM1_ESM.docx]

**Addition File for**

**Effects of PM_2.5_ and gases exposure during prenatal and early-life on autism–like phenotypes in male rat offspring**

Baharan Emam^a^, Abbas Shahsavani^b,a^*, Fariba Khodagholi^c^, Saeed Motesaddi Zarandi^a^, Philip K. Hopke^d,e^, Mostafa Hadei ^f,g^, Hamidreza Behbahani^c^, Maryam Yarahmadi^h^

^a^ Department of Environmental Health Engineering, School of Public Health and Safety, Shahid Beheshti University of Medical Science, Tehran, Iran

^b^ Environmental and Occupational Hazards Control Research Center, Shahid Beheshti University of Medical Sciences, Tehran, Iran

^c^ Neuroscience Research Center, Shahid Beheshti University of Medical Sciences, Tehran, Iran

^d^ Department of Public Health Sciences, University of Rochester School of Medicine and Dentistry, Rochester, NY 14642 USA

^e^ Center for Air Resources Engineering and Science, Clarkson University, Potsdam, NY 13699 USA

^f^ Department of Environmental Health Engineering, School of Public Health, Tehran University of Medical Sciences, Tehran, Iran

^g^ Students' Scientific Research Center (SSRC), Tehran University of Medical Sciences, Tehran, Iran

^h^ Center of Environmental and Occupational health, Ministry of Health and Medical Education, Tehran, Iran

***Corresponding author:** Abbas Shahsavani

Environmental and Occupational Hazards Control Research Center, Shahid Beheshti University of Medical Sciences, Tehran, Iran.

E-mail: ashahsavani@gmail.com; Tel: +989102006560

**Table S1.** Concentration of Metals bound PM2.5 in exposure period

| Heavy metals | Mean | SD |
| --- | --- | --- |
| Al | 35.01 | 16.57 |
| Cu | 1.04 | 4.53 |
| Fe | 0.75 | 0.12 |
| Mn | 0.12 | 0.07 |
| Ca | 56.5 | 4.21 |
| Cd | 0.59 | 3.64 |
| Cr | 0.32 | 0.22 |
| Na | 3.02 | 3.26 |
| Ni | 0.31 | 0.1 |
| Pb | 0.23 | 0.19 |
| As | 0.06 | 0.1 |
| V | 0.04 | 0.1 |
| Zn | 0.19 | 0.1 |
| Sum | 98.18 | 33.21 |

**Table S2.** Concentration of 16-PAHs bound PM_2.5_ in exposure period

| PAHs | Mean | SD |
| --- | --- | --- |
| Naphtalene | 4.42 | 4.69 |
| Acenaphtylen | 2.85 | 0.88 |
| Acenaphten | 2 | 0.74 |
| Florene | 3.84 | 1 |
| Phenanthrene | 6.76 | 2.29 |
| Anthracene | 2.88 | 1.41 |
| fluorantene | 2.44 | 0.95 |
| Pyrene | 3.12 | 1.05 |
| Benzo(a)Anthracene | 2.25 | 1.48 |
| Chrysene | 2.56 | 1.18 |
| Benzo(b)Fluoranthene | 2.59 | 1.08 |
| Benzo(K)Fluoranthene | 4.38 | 1.24 |
| Benzo(a)Pyrene | 1.33 | 0.69 |
| Dibenzo(a,h)Anthracene | 1.7 | 0.82 |
| Benzo(g,h,i)perylene | 1.67 | 0.98 |
| Indeno(1,2,3-cd) pyrene | 1.09 | 0.54 |
| Sum | 45.88 | 21.02 |
